# Investigation of monosodium glutamate content in flavors, seasonings, and sauces from local markets in Turkey

**DOI:** 10.1002/fsn3.4406

**Published:** 2024-08-11

**Authors:** Yusuf Katrancı, Aleyna Aydemir, Beray Kızılkaya, Gizem Yıldırım Baştemur, Sabriye Percin Ozkorucuklu

**Affiliations:** ^1^ Department of Molecular Biology and Genetics, Faculty of Science Istanbul University Istanbul Turkey; ^2^ Programme of Molecular Biotechnology and Genetics Institute of Graduate Studies in Sciences, Istanbul University Istanbul Turkey

**Keywords:** food additives, method validation, monosodium glutamate, RP‐HPLC

## Abstract

Monosodium glutamate (MSG) is a flavor‐enhancing compound used to elevate the flavor profiles of various foods. This flavor enhancer is the sodium salt of L‐glutamic acid and is widely used in foods, such as flavorings, seasonings, sauces, and instant soups. The potential health effects of MSG consumption, particularly the health issues that excessive consumption could lead to, have been the focus of social concerns. It is stated that excessive consumption of MSG can cause cardiovascular diseases, obesity and diabetes, kidney damage, hypertension, anxiety, and memory impairment. The maximum permissible amount of MSG in foods is set at 10 g/kg according to the Turkish Food Codex. The aim of this study is to develop an appropriate method for analyzing the MSG content within the various food samples like flavorings, seasonings, and spices sold in Turkish local markets. The validation parameters of the developed method were examined and it was found that the developed method corresponds to the recommended values. The limit of detection and the limit of quantitation values were calculated as 4.78 ng/mL and 15.93 ng/mL, respectively. Recovery % was determined to be 100.96% in intra‐day and 132.22% in inter‐day analyses for the precision of the method. The results compared to the values specified in the Turkish Food Codex Food Additives Regulation and samples that purportedly did not contain MSG on their labels were found to contain MSG.

## Graphical abstract

The aim of this study is to develop an appropriate method for analyzing the monosodium glutamate content within the various food samples like flavorings, seasonings, and spices sold in Turkish local markets.

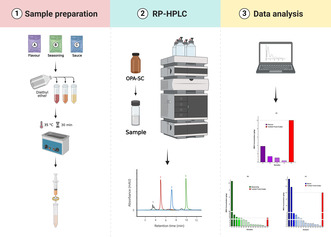

## 1 INTRODUCTION

Food additives have been utilized from ancient times to the modern age with the purpose of preserving, enhancing, or modifying the characteristics and nutritional value of foods (Abdulmumeen et al., 2012; Kökmen Seyirci & Çağ, 2018). The widespread usage of food additives has become increasingly prevalent due to various reasons, such as the rapid growth of the global population and the rational utilization of food resources to decelerate food spoilage and extend shelf life (Erden Çalışır & Çalışkan, 2003).

Flavor enhancers constitute a category of food additives employed to enrich the aroma and provide a complex taste profile in foods (Vasilaki et al., 2022). Monosodium glutamate (MSG) is a white, crystalline flavor enhancer known as the sodium salt of glutamic acid (Kayode et al., 2023). In addition to the four primary tastes, this compound possesses the fifth basic taste, known as umami (Ault, 2004; Ikeda, 2002). Despite the acknowledged existence of the umami taste, research on how it enhances the overall flavor of food remains unexplored (Ma et al., 2023; Yamamoto & Inui‐Yamamoto, 2023). MSG can be found in various food products, including crackers, processed meats, instant soups, meat and chicken bouillons, spices, seasonings, chips, flavorings, sauces, and cheeses (Bayram et al., 2023; Cebi et al., 2018).

Concerns related to MSG consumption are primarily associated with health implications and food safety. In 1986, a series of symptoms, such as widespread pain, palpitations, and numbness, were reported by individuals who had dined in Chinese restaurants across various regions. Research conducted in the following years proposed a potential connection between these symptoms and MSG (Kwok, 1968; Morselli & Garattini, 1970; Schaumburg, 1968). Recent research on monosodium glutamate (MSG) have investigated its potential health effects, suggesting that MSG may be linked to genotoxicity, reductions in erythrocyte counts, increased oxidative stress, weight gain, learning difficulties, and adverse effects on embryonic development (Ashaolu et al., 2011; Ataseven et al., 2016; Bölükbaş & Öznurlu, 2023; Duran & Aki, 2017; López‐Miranda et al., 2015; Sauganth Paul et al., 2012; Ün & Büyükuslu, 2018).

The contradictory and concerning situations related to the consumption of MSG have prompted many authorities to implement regulations in the use of MSG: Respected organizations such as the United States Food and Drug Administration (FDA, 1995) and the Joint FAO/WHO Expert Committee on Food Additives (JECFA) (WHO, 1987) have conducted studies on the safe use of MSG. European Food and Safety Authority (EFSA) (Mortensen et al., 2017), for instance, has designated “Acceptable Daily Intake (ADI)” for glutamic acid and its salts. In addition, the European Parliament and Council Directive No 95/2/EC (Directive, 1995) and the Turkish Food Codex Regulation on Food Additives Annex‐II (TFC, 2013) have limited the use of this substance, identified by the code E621, to a maximum of 10 g/kg.

The presence and quantity of MSG in food has been a matter of significance. There are several methods for determination of MSG (Nguyen Thuy et al., 2020), including high‐performance thin‐layer chromatography (HPTLC) (Krishna et al., 2010), flow injection analysis (FIA) (Oliveira et al., 2001), multiwalled carbon nanotube‐based potentiometry (Anirudhan & Alexander, 2015), and high‐performance liquid chromatography (HPLC) with various detectors (such as ultraviolet (UV), fluorescence, and diode array detector (DAD)) (Er Demirhan et al., 2015; Kamal et al., 2023; Lateef et al., 2012; Populin et al., 2007). Among these techniques, HPLC is favored for its high separation efficiency, good selectivity, and heightened detection sensitivity. High‐performance liquid chromatography (HPLC) has emerged as a widely employed method of separation in contemporary applications, owing to its capability to effectively separate temperature‐sensitive substances, facilitating efficient separation and swift analysis.

This study aimed to determine the content of monosodium glutamate (MSG) in food samples using reversed phase‐HPLC (RP‐HPLC) method. First, the ratio of MSG‐derivatizing agent, flow rate, column temperature, and mobile phase composition were optimized. Second, parameters, such as system suitability, linearity, and sensitivity, were evaluated. Finally, MSG content in a large number of purchased flavors, seasonings, and sauces was analyzed, and the results were compared to the specified 10 g/kg value in the Turkish Food Codex (TFC). This study is associated with the potential to contribute to human health, maintain label consistency in products, and provide a contribution for analyzing institutions.

## 2 EXPERIMENTAL

### 2.1 Standards and reagents

Monosodium glutamate, L‐glutamic acid, OPA‐SC (o‐phthaldialdehyde reagent‐solution complete), uracil, formic acid, methanol, diethyl ether, and hydrochloric acid (HCL) were purchased from Sigma‐Aldrich. All utilized chemicals were of analytical quality, and the solvents were of HPLC grade. The Milli‐Q water purification system was utilized for the production of purified ultrapure water (Millipore, Bedford, MA, USA). Before HPLC analysis, membrane filters (Sartorius model 0.45‐μm polytetrafluoroethylene (PTFE) filter) were employed to filter the mobile phase, standards, and samples.

### 2.2 HPLC instruments and conditions

The analyses were performed on a Shimadzu HPLC system that was equipped with a pump (LC‐10AD VP), an autosampler (SIL‐20A), a column oven (CTO‐10AS), a degassing unit (DGU‐20A), and a diode array detector (SPD‐M20A). Chromatographic analyses were carried out on the Mediterranea Sea 18 (15 × 0.46 cm, 5 μm) column by gradient program using mobile phase A (the deionized water containing 0.5% formic acid) and mobile phase B (methanol). The column was maintained at a constant temperature of 20°C using a column thermostat and the flow rate was kept at 0.8 mL/min. The wavelength for the DAD was configured to 225 nm. The injection volume for every sample and standard was set at 20 μL, and the injection time was adjusted to 25 min.

### 2.3 Preparation of stock solution

The standard MSG stock solution at a concentration of 2500 μg/mL was prepared by dissolving in deionized water and kept at +4°C. The standard solutions for the calibration curve were prepared by diluting the stock solution with deionized water, resulting in the attainment of the appropriate lower concentrations (0.25, 0.5, 1, 2.5, and 5 μg/mL) of the standard MSG solutions. L‐Glutamic acid was dissolved in deionized water to prepare a 250 μg/mL solution for its utilization as a standard in the system suitability test (SST), and uracil was dissolved in methanol to prepare 20 μg/mL solution for determining the dead time (*t*_0_). These solutions that were prepared underwent filtration using a syringe filter and were appropriately stored at 4°C.

### 2.4 Preparation of sample extracts

A total of 33 different food samples comprising 4 flavors, 15 seasonings, and 14 sauces were purchased from various local markets in Istanbul, Turkey, and stored in a dry and cool place. All samples were carefully weighed to be 0.1 g each using a precision balance. Subsequently, 10 mL of 0.1 M HCl solution was added, and the mixture was subjected to sonication in an ultrasonic bath at 50°C for 30 min. During the extraction process, 5 mL of extract was taken, and an equal volume of diethyl ether was added to it, ensuring thorough homogenization of the mixture. Subsequently, the removal of fatty acids from this mixture was achieved through the use of an evaporator. The extracts were subsequently filtered through a 0.45‐μm microfilter and stored at +4°C.

Pre‐column derivatization was required for both the standard MSG and all samples. All samples and MSG standard were derivatized with OPA‐SC with a dilution ratio of 1:9 (v:v) (sample: OPA‐SC). After a 2‐min incubation period, three repeated injections of standards and samples into the RP‐HPLC system were performed.

## 3 RESULTS AND DISCUSSION

### 3.1 Optimization of RP‐HPLC method

The determination of the chromatographic method is one of the most crucial stages in the analysis of samples. The developed method should be efficient, with a short analysis time, and well‐defined peaks that are symmetric and narrow. Considering these criteria, optimum conditions were investigated to determine the most effective method for the analysis of monosodium glutamate (MSG). In this developed method, OPA‐SC ratios, the percentage of formic acid in the mobile phase A, flow rate, and column temperature were optimized.

Analysis of substances within the 200–800 nm wavelength range requires the presence of chromophore groups. Since these groups are not intrinsically present in the structure of MSG, a pre‐column derivatization procedure using OPA‐SC was employed to facilitate the addition of chromophore groups into the MSG structure (Er Demirhan et al., 2015; Kamal et al., 2023; Soyseven et al., 2021). The MSG:OPA‐SC (v/v) mixtures in ratios of 1:5, 1:6, 1:7, 1:9, 1:10, 1:12, and 1:15 were investigated to determine the optimum MSG to OPA‐SC ratio. The selection of the mobile phase is a critical determinant in the efficient compound detection. In gradient system for this study, methanol was employed as mobile phase B, whereas mobile phase A comprised a deionized water and formic acid mixture. For comparison purposes, five different percentage levels of formic acid (0.0%, 0.1%, 0.25%, 0.5%, and 1.0%) present in the mobile phase A were investigated. Also, the impact of varying flow rates (0.5, 0.6, 0.8, 1.0, and 1.2 mL/min) and column temperatures (20, 25, 30, and 35°C) was assessed.

Consequently, when parameters, such as peak symmetry, peak areas, and analysis time, were evaluated, the optimal conditions were a 1:9 ratio of OPA‐SC to MSG, a mobile phase A containing 0.5% formic acid–deionized water mixture, 0.8 mL/min. flow rate, and 20°C column temperature. The chromatographic data of the MSG under optimum conditions are presented in Table 1.

**TABLE 1:** The values of capacity factor, selectivity, and resolution factor of the MSG.

Compound	*k*_₂_	*α*	(*α* − 1)/α	(*k*_₂_)/(*k*_₂_ + 1)	1/4 √N	*R*_*s*_
Monosodium glutamate	6.49	1.29	0.23	0.87	51.00	10.02

### 3.2 Validation of RP‐HPLC method

#### 3.2.1 System suitability tests

System suitability tests play a crucial role in validating the robustness and reproducibility of an analytical method. The results of these tests help determine under what conditions an analysis can be used reliably and ensure the consistency of analytical data (ICH, 2005). In the system suitability study, various parameters, such as retention time, capacity factor, selectivity, theoretical plates, tailing factor, resolution, and the relative standard deviation (%RSD), were examined. Five replicate injections were carried out using a standard MSG solution with a concentration of 1 μg/mL. The determination of the capacity factors of the compounds involved selecting uracil as the reference for dead time (*t*_0_). The parameters related to system suitability, as specified in Table 2, conform to the specified criteria, confirming the adequacy of the system. The findings from the assessment of system suitability affirm that the chosen chromatographic parameters are appropriate for analyzing the MSG.

**TABLE 2:** System suitability test results.

Compound	RT (min)	Capacity factor (*k*)	Selectivity (*α*)	Theoretical plates (*N*)	Tailing factor (*T*_*f*_)	Resolution (*R*_*s*_)	% RSD (min)
Recommended values	**–**	1–10	≥1.15	>2000	≤2	≥1.5	≤1
Monosodium glutamate	22.13	6.49	1.29	41,610	1.24	10.02	0.07

#### 3.2.2 Linearity

Linearity is the ability of a method to obtain a result that is directly proportional to the concentration of the analyte in the sample (Akpınar et al., 2023). The method's linearity was evaluated by performing five replicate injections of five known analyte concentrations within the 0.25–5 μg/mL range. The linear regression analysis yielded a correlation coefficient of 0.9999 for MSG, indicating good linearity in the method that was optimized following the International Council for Harmonisation of Technical Requirements for Pharmaceuticals for Human Use (ICH) guidelines (ICH, 1996).

#### 3.2.3 Sensitivity

The values of the limit of detection (LOD) and limit of quantification (LOQ) found in the method can be defined as the lowest level that the method can determine and the lowest level that can be quantified, respectively (Akpınar et al., 2023; Gavage et al., 2022). The LOD and LOQ values were determined through the normalization of the signal‐to‐noise ratio (S/N) to 3 for LOD and S/N to 10 for LOQ, utilizing the standard deviation (SD) of the response and the slope of the calibration curve. Thus, the LOD and LOQ values for MSG were calculated as 4.78 ng/mL and 15.93 ng/mL, respectively. These findings suggested that the method offered sufficient sensitivity. It was determined that the LOD and LOQ values were much lower in the literature (Er Demirhan et al., 2015; Soyseven et al., 2021; Soyseven & Arli, 2022; Wang et al., 2019).

#### 3.2.4 Precision

Precision is a measure of the reproducibility of the developed method, indicating how closely the obtained results were under the same conditions. The determination of the precision parameter is carried out through intra‐day and inter‐day studies (Cebi et al., 2018). In intra‐day and inter‐day precision studies, experiments were carried out using a reference concentration of 1 ppm (part per million) within the calibration range for monosodium glutamate standard. Intraday precision was assessed by conducting a minimum of nine injections on the same day, while inter‐day precision was evaluated by performing injections on consecutive days. Precision assessment relies on the relative standard deviation (%RSD) of test outcomes, with a requirement that this value remains below 2%. Recovery % was determined to be 100.96 in intra‐day and 132.23 in inter‐day analyses for the precision of the method. The relatively high %RSD value identified in the inter‐day study can be attributed to the potential degradation of the interaction between the MSG standard and the OPA‐SC derivative over time (Soyseven et al., 2021). The values obtained from the precision studies are presented in Table 3.

**TABLE 3:** Results of intra‐day and inter‐day values of monosodium glutamate.

	Concentration (mg/L, ppm)	Recovery (%) ± SD	RSD (%)
Intra‐day	1	100.96 ± 0.42	0.42
Inter‐day	1	132.23 ± 1.32	1.00

### 3.3 Analysis of food samples

The analysis of monosodium glutamate content in diverse brands of flavors, seasonings, and sauces, regardless of their stated MSG content, was developed and validated using the RP‐HPLC method. The quantitative determination of monosodium glutamate for each sample has been calculated using the regression equation obtained from the calibration curve. Each sample was analyzed in five replicates, and the amounts of MSG found in food samples are given in Table 4.

**TABLE 4:** The RP‐HPLC results of the MSG present in 33 food samples.

Number	Group	Sample name	Concentration of MSG (g/kg) ± SD
1	A1	Flavor 1^a^	3.81 ± 0.83
2	A2	Flavor 2^a^	1.37 ± 0.10
3	A3	Flavor 3^a^	1.05 ± 0.05
4	A4	Flavor 4^a^	0.37 ± 0.14
5	B1	Seasoning 1^b^	102.88 ± 10.36
6	B2	Seasoning 2^b^	31.50 ± 9.40
7	B3	Seasoning 3^b^	16.19 ± 0.21
8	B4	Seasoning 4^b^	10.40 ± 3.29
9	B5	Seasoning 5^b^	8.62 ± 0.07
10	B6	Seasoning 6^a^	3.44 ± 1.35
11	B7	Seasoning 7^a^	3.09 ± 0.80
12	B8	Seasoning 8^a^	1.98 ± 0.44
13	B9	Seasoning 9^a^	1,05 ± 0.06
14	B10	Seasoning 10^a^	0,95 ± 0.16
15	B11	Seasoning 11^a^	0.82 ± 0.22
16	B12	Seasoning 12^a^	0.78 ± 0.06
17	B13	Seasoning 13^a^	0.68 ± 0.04
18	B14	Seasoning 14^a^	0.49 ± 0.02
19	B15	Seasoning 15^b^	0.08 ± 0.12
20	C1	Sauce 1^b^	11.06 ± 0.52
21	C2	Sauce 2^b^	10,44 ± 0.59
22	C3	Sauce 3^a^	7.58 ± 2.15
23	C4	Sauce 4^b^	6.32 ± 2.36
24	C5	Sauce 5^b^	4.21 ± 0.79
25	C6	Sauce 6^b^	3.19 ± 0.01
26	C7	Sauce 7^a^	1.77 ± 0.14
27	C8	Sauce 8^a^	1.10 ± 0.03
28	C9	Sauce 9^a^	0.95 ± 0.11
29	C10	Sauce 10^a^	0.94 ± 0.02
30	C11	Sauce 11^b^	0.90 ± 0.15
31	C12	Sauce 12^b^	0.63 ± 0.06
32	C13	Sauce 13^a^	0.60 ± 0.01
33	C14	Sauce 14^b^	0.20 ± 0.08

^a^ It is indicated on the labels of these products that there is no MSG content in the product.

^b^ It is indicated on the labels of these products that there is MSG content in the product.

**FIGURE 1:**
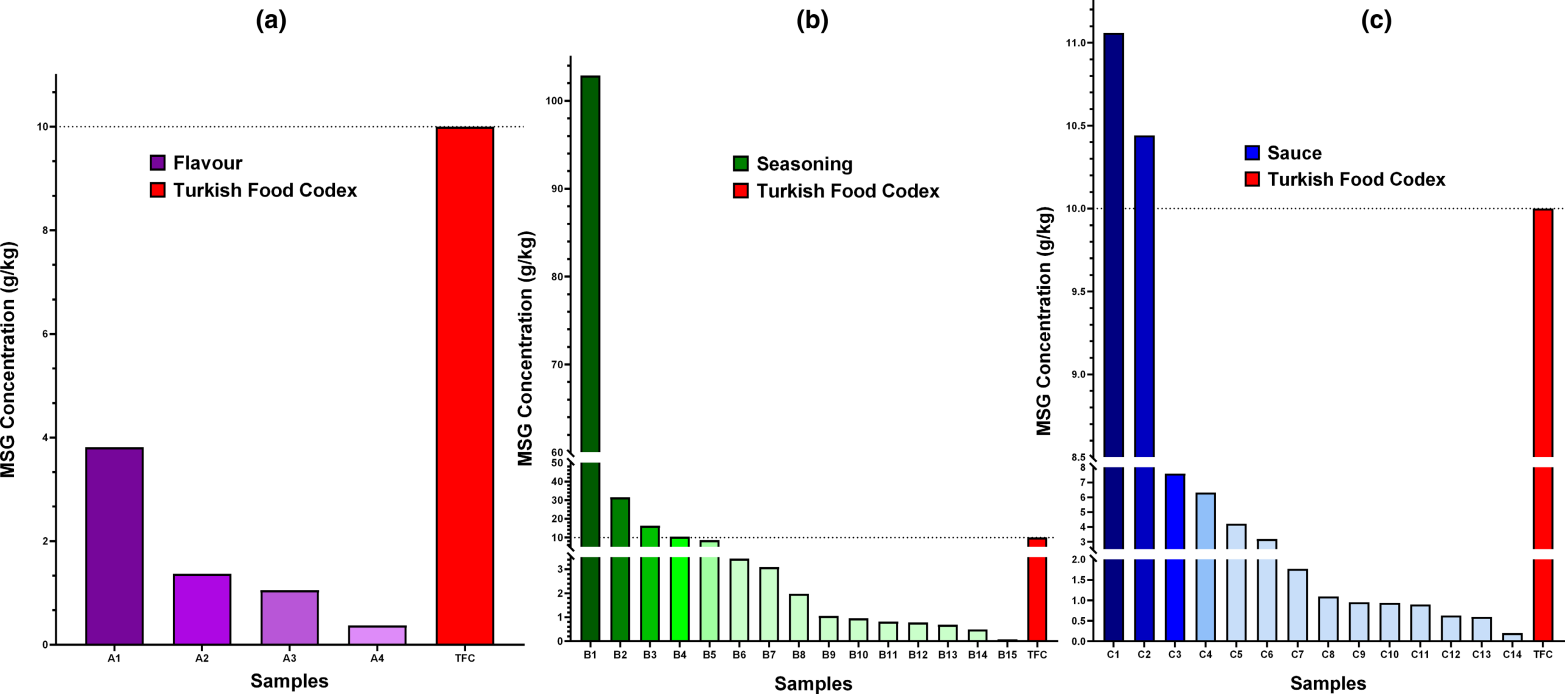
Comparative analysis of monosodium glutamate concentrations (gram per kilogram (g/kg)) in various (a) flavors, (b) seasonings, and (c) sauce food samples according to the Turkish Food Codex (TFC).

According to Table 4, the levels of monosodium glutamate (MSG) in 33 samples, with and without information on the presence of MSG, were found to be different. The MSG values in the seasoning samples were found to be between 0.08 and 102.88 g/kg, as seen in Figure 1, and higher values were obtained compared to the other two sample groups. Seasoning samples were followed by sauces with amounts in the range of 0.20–11.06 g/kg and flavors with amounts in the range of 0.37–3.81 g/kg. Of the samples subjected to quantitative determination, a total of six samples, including four seasonings and two sauces, were found to contain MSG levels higher than the 10 g/kg value specified in the Turkish Food Codex Food Additives Regulation. As a result of the determination, MSG was found in 19 samples that were not stated to contain MSG.

Lateef et al. (2012) utilized the HPLC‐UV (ultraviolet) method to detect monosodium glutamate (MSG) in various commercial Pakistani spices, revealing concentrations ranging from 27 to 88 g/kg. In a study by Er Demirhan et al. (2015), the HPLC–DAD method was employed to determine MSG levels in 122 chicken and beef stock cubes from four different brands, resulting in concentrations ranging from 7.2 ± 0.1 to 14.6 ± 0.2 g/kg. Cebi et al. (2018) conducted analyses on chips, taste cubes, sauces, and soup samples using the liquid chromatography with tandem mass spectrometry (LC–MS/MS) method to quantify free glutamic acid and analyze monosodium glutamate. The results indicated MSG content in the range of 0.01–15.39 g per 100 g of food samples. Ayad et al. (2021) employed the HPLC–UV (ultraviolet)/DAD method to determine MSG levels in 50 different meat products, obtaining values within the range of 1.47 ± 0.85–3.95 ± 0.51 mg/g. Additionally, Soyseven et al. (2021) found MSG concentrations ranging from 0.09 to 120.80 g/kg in different sample groups using the HPLC‐UV (ultraviolet)/DAD method. Huang et al. (2021) used the HPLC‐UV (ultraviolet) method to detect monosodium glutamate (MSG) in a selection of 20 pungent spices. Their findings revealed MSG concentrations ranging from 0.2 to 37.1 g MSG/100 g across the different spices. A review of the literature revealed that the values identified in our study are consistent with those found in previous studies.

**FIGURE 2:**
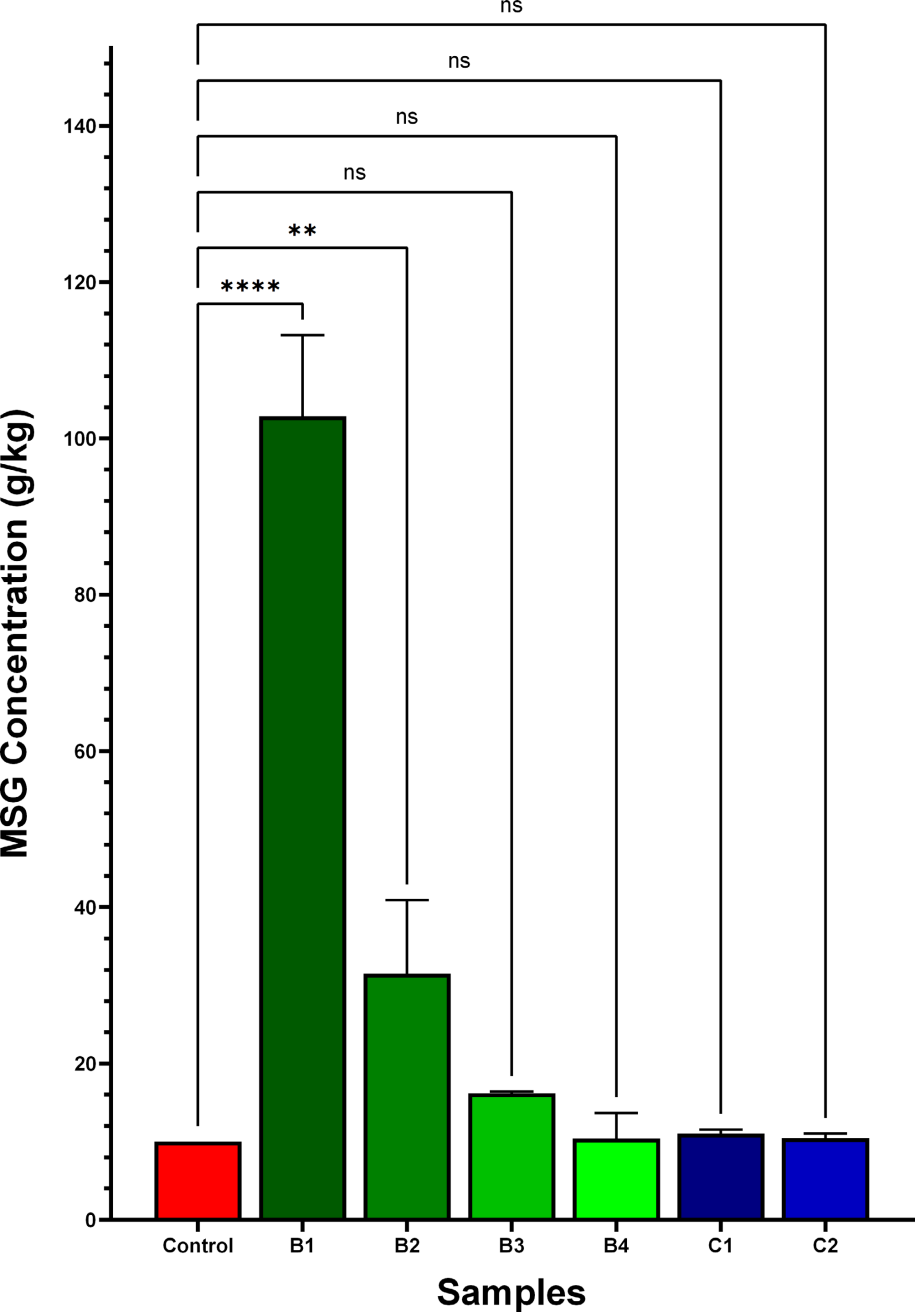
Comparative analysis of the significance levels of monosodium glutamate concentrations (gram per kilogram (g/kg)) between the excessive amounts found according to Turkish Food Codex (TFC). The results are expressed as a percentage of the control by one‐way analysis of variance (ANOVA) followed by the Dunnett test (≥.05 (ns), .01–.05 (*), .001–.01 (**), .0001–.001 (***), and < .0001(****) vs. Control).

The quantitative results of the samples were analyzed by using GraphPad Prism 10.2.0 software, applying one‐way analysis of variance (ANOVA) followed by Dunnett's test for statistical analysis. The results of this analysis are shown in Figure 2. All data were expressed as the mean values ± standard deviation of triplicate results. Statistical significance was determined based on predefined thresholds: (≥.05 (ns), .01 to .05 (*), .001 to .01 (**), .0001 to .001 (***), and <.0001 (****)), which were categorized as not significant, significant, very significant, and extremely significant, respectively. When comparing the six samples with high amounts of MSG content according to the Turkish Food Codex, a significant difference in the level of significance was observed in B1 and B2. However, there was no significant difference in the accepted significance level among the other samples B3, B4, C1, and C2. These findings suggest that the consumption of two samples (B1 and B2) with monosodium glutamate (MSG) levels exceeding the Turkish Food Codex is not acceptable compared to the other four samples.

## 4 CONCLUSION

In this study, a simple, efficient, and reliable RP‐HPLC method has been developed and validated for the determination of monosodium glutamate (MSG) in a total of 33 food samples, including flavors, seasonings, and sauces. The developed method involved the optimization of flow rate, column temperature, mobile phase composition, and MSG‐derivatization agent ratios. Subsequently, the system suitability test, linearity, sensitivity, and precision parameters were investigated to complete the method validation. Using this newly developed and validated method, the analysis of MSG in food samples was successfully conducted. The results indicate that six samples, whether labeled with MSG information or not, exceeded the limits set by the European Directive and Turkish Food Codex for MSG content. Additionally, when comparing sample groups, seasoning products showed a higher MSG content compared to others, while flavor group samples exhibited the least MSG content. This developed method plays a crucial role in the regulation of MSG in the food industry, raising consumer awareness, and contributing to healthy food production, thus playing a significant role in safeguarding consumer health.

## AUTHOR CONTRIBUTIONS

**Yusuf Katrancı:** Data curation (equal); software (equal); visualization (equal). **Aleyna Aydemir:** Data curation (equal); software (equal); visualization (equal). **Beray Kızılkaya:** Formal analysis (equal); investigation (equal); writing – original draft (equal). **Gizem Yıldırım Baştemur:** Investigation (equal); methodology (equal); validation (equal); writing – review and editing (equal). **Sabriye Percin Ozkorucuklu:** Conceptualization (equal); methodology (equal); project administration (equal); supervision (equal); writing – review and editing (equal).

## FUNDING INFORMATION

This study was funded by the Scientific Research Projects Coordination Unit of Istanbul University (Project number: FLO‐2022‐39584).

## CONFLICT OF INTEREST STATEMENT

The authors declare that they have no conflict of interest.

## Data Availability

The data supporting this study's findings are available on request from the corresponding author.
